# Testis-specific peroxiredoxin 4 variant is not absolutely required for spermatogenesis and fertility in mice

**DOI:** 10.1038/s41598-020-74667-9

**Published:** 2020-10-21

**Authors:** Takujiro Homma, Toshihiro Kurahashi, Naoki Ishii, Nobuyuki Shirasawa, Junichi Fujii

**Affiliations:** 1 Department of Biochemistry and Molecular Biology, Graduate School of Medical Science, Yamagata University, 2-2-2 Iidanishi, Yamagata, 990-9585 Japan; 2 Department of Rehabilitation, Faculty of Medical Science and Welfare, Tohoku Bunka Gakuen University, Sendai, 981-8551 Japan; 3 Present Address: Department of Cellular Regenerative Medicine, Graduate School of Medical Science, Kyoto Prefectural University of Medicine, Kyoto, Japan

**Keywords:** Biochemistry, Molecular biology

## Abstract

PRDX4, a member of peroxiredoxin family, is largely concentrated in the endoplasmic reticulum (ER) and plays a pivotal role in the redox relay during oxidative protein folding as well as in peroxidase reactions. A testis-specific PRDX4 variant transcript (PRDX4t) lacks the conventional exon 1, which encodes the signal peptide that is required for entry into the ER lumen, but instead carries alternative exon 1, which is transcribed from the upstream promoter in a testis-specific manner and results in the PRDX4t protein being localized in the cytosol. However, the potential roles of PRDX4t in male genital action remain unknown. Using a CRISPR/Cas9 system, we first disrupted the testis-specific promoter/exon 1 and generated mice that were specifically deficient in PRDX4t. The resulting PRDX4t knockout (KO) mice underwent normal spermatogenesis and showed no overt abnormalities in the testis. Mating PRDX4t KO male mice with wild-type (WT) female mice produced normal numbers of offspring, indicating that a PRDX4t deficiency alone had no effect on fertility in the male mice. We then generated mice lacking both PRDX4 and PRDX4t by disrupting exon 2, which is communal to these variants. The resulting double knockout (DKO) mice were again fertile, and mature sperm isolated from the epididymis of DKO mice exhibited a normal fertilizing ability in vitro. In the meantime, the protein levels of glutathione peroxidase 4 (GPX4), which plays an essential role in the disulfide bond formation during spermatogenesis, were significantly increased in the testis and caput epididymis of the DKO mice compared with the WT mice. Based on these results, we conclude that the disruption of the function of PRDX4t in the spermatogenic process appears to be compensated by other factors including GPX4.

## Introduction

Peroxiredoxin (PRDX) catalyzes the reductive removal of hydrogen peroxides using thioredoxin as an electron donor and also plays multiple roles in redox reactions including intracellular signaling^1,2^. Among the members of the PRDX family, PRDX4 is expressed systemically and is translated with an additional N-terminal extension consisting of a signal peptide that is responsible for translocation into the lumen of the endoplasmic reticulum (ER)^3,4^. In the ER lumen, ER oxidoreductin 1 (ERO1) introduces a disulfide bond into protein disulfide isomerase (PDI) using molecular oxygen as the electron acceptor and releases hydrogen peroxide as a byproduct^5^. In the meantime, PRDX4 uses hydrogen peroxide to oxidize sulfhydryl groups to disulfide groups in the PDI^6,7^. PDI, in turn, introduces disulfide bonds in nascent target proteins. While a single deficiency of ERO1 or PRDX4 shows no apparent phenotype, a double deficiency of them causes the development of atypical scurvy due to aberrant collagen synthesis in mice^8^. Thus, PRDX4 simultaneously exerts two beneficial functions in the ER lumen; acceleration of the proper oxidative folding of nascent proteins and the detoxification of hydrogen peroxide, and prevents the ER stress^9,10^.

The PRDX4 protein is present in a single molecular size in most tissues, but an extra protein with a larger molecular size is present only in sexually mature testes^11,12^. This is caused by the expression of a splicing variant only in sexually mature testes and is hereafter referred to as the *Prdx4t* gene^9,13^. The *Prdx4t* gene is transcribed from the alternative promoter/exon 1 that is localized upstream from the promoter/exon 1 which is responsible for the systemic transcription in the gene^13^. The translated PRDX4t protein lacks the signal peptide and hence is localized in the cytosol in spermatids of sexually mature testes. The genetic knockout (KO) of the systemic promoter/exon1 of the *Prdx4* gene results in the complete inhibition of PRDX4 production in most tissues in mice^14^, but the PRDX4t protein continues to be present in a certain stage of spermatogenic cells, although to a slightly decreased extent^9,13^. This appears to be associated with a delay in the sexual maturation, although adult PRDX4 KO male mice are fertile and show nearly normal phenotypes in most tissues^14^. Essential cysteine required for catalysis is encoded by the communal exon 2 between *Prdx4* and *Prdx4t*. The mature form of PRDX4, after proteolytic removal of the N-terminal signal peptide, and PRDX4t possess the ability to reduce hydrogen peroxides at similar rates^15,16^.

During the course of spermatogenesis, histones in chromatin are converted to transition proteins as intermediary proteins and finally to protamines^17^, which is a prerequisite process for sperm function as well as structural rigidity^18,19^. The protamines of primates and rodents contain multiple cysteine residues that are oxidized to form disulfide bridges. This formation furnishes chromatin for resistance against oxidative stress and compacts the sperm nucleus, suggesting that enzymes responsible for the crosslinking could play a pivotal role in producing functional spermatozoa. While phospholipid hydroperoxide glutathione peroxidase (GPX4) could be responsible for the maturation of spermatozoa via supporting disulfide bond formation^20^, additional enzymes also appear to be involved in the reaction and provide support for sperm potency. Given the role of PRDX4 in oxidative protein folding in the ER, it is possible that PRDX4t is also involved in the introduction of disulfide bridges in protamines during oxidative chromatin packaging in sperm^20^.

In the current study, we generated mice with a genetic depletion of the PRDX4t expression via CRISPR/Cas9-mediated genome editing in order to reveal potential roles of this PRDX4t in the male reproduction and spermatogenesis. Our results suggest that PRDX4t expression is not essential for mouse fertility or spermatogenesis.

## Materials and methods

### Generation and maintenance of genome editing mice

PRDX4t KO or PRDX4/PRDX4t DKO mice were generated using the CRISPR/Cas9 system at the Research Laboratory for Molecular Genetics at Yamagata University. For the CRISPR RNAs (crRNA) design, CRISPRdirect (https://crispr.dbcls.jp/) was used to select unique target sites throughout the mouse genome. All of the crRNAs, trans-activating crRNA (tracrRNA), and the Cas9 protein were microinjected into fertilized zygotes of C57BL/6N female mice (Japan SLC, Hamamatsu, Japan). The crRNAs and tracrRNA were chemically synthesized and HPLC-purified by Integrated DNA Technologies (Coralville, IA, USA) and are listed in Supplementary Table S1. Mutations in *Prdx4* gene were confirmed by Sanger sequence analyses (ABI 3500 Genetic Analyzer; Thermo Fisher Scientific, Waltham, MA, USA). The genotype of each mouse was confirmed by PCR-based analysis using the primers listed in Supplementary Table S2. PRDX4t KO or PRDX4/PRDX4t DKO male mice were produced by mating KO/DKO males with heterozygous KO/DKO females and were used for the phenotypic analyses in parallel with age-matched wild-type (WT) littermates as a control group (the *Prdx4* gene is linked to X-chromosome). Adult (11–15 week old) male mice were used throughout the study unless otherwise stated. The animal room climate was maintained under specific pathogen-free conditions at a constant temperature of 20–22 °C with a 12 h alternating light–dark cycle, and food and water were available ad libitum. Animal experiments were performed in accordance with the Declaration of Helsinki under the protocol approved by the Animal Research Committee of Yamagata University.

### Histological and immunohistochemical analysis

Tissues were dissected and fixed in modified Davidson’s fluid^21^ overnight at 4 °C followed by embedding in paraffin. Sections (5 μm in thickness) were stained with H&E (hematoxylin–eosin) or PAS (periodic acid-Schiff). Immunohistochemical staining was performed using rabbit antibodies against total PRDX4^14^ or PRDX4t^9^ as described previously^22^. The tissue sections were first reacted with primary antibodies followed by a peroxidase-labeled goat anti-rabbit IgG. Specific immunolabeling was visualized using a chromogen, 3,3′-diaminobenzine (Dojindo, Tokyo, Japan). The images were obtained with a BZ-X700 microscope (KEYENCE, Osaka, Japan).

For immunofluorescence analyses, the antibodies that were bound were visualized using Alexa Fluor 488-conjugated or Alexa Fluor 568-conjugated goat anti-rabbit IgG antibodies (Thermo Fisher Scientific) under a confocal laser scanning microscope (PASCAL; Carl Zeiss, Oberkochen, Germany).

### Counting sperm numbers

The cauda epididymis was dissected from the mice, transferred to PBS, and minced into small pieces. After incubation at room temperature for 15 min, the released spermatozoa were suspended to produce a homogeneous mixture. Sperm numbers were counted under a light microscope.

### Assessing reproductive ability of male mice

The fertilizing ability of the male mice was examined as described previously with minor modifications^14^. Individual male mice at 12-weeks of age were cohabitated with a sexually mature WT female mouse with C57BL/6N backgrounds until pregnancy was attained. Upon delivery, the number of pups for each mouse was counted.

### Western blot analysis

Tissues were homogenized with a glass-teflon homogenizer in lysis buffer (50 mM Tris–HCl pH 7.5, 150 mM NaCl, 1% NP-40, 0.5% sodium deoxycholate, 0.1% SDS), supplemented with a protease inhibitor cocktail (Sigma-Aldrich, St. Louis, MO, USA; P8340). The lysate was centrifuged at 15,000 × *g* for 10 min in a microcentrifuge. Protein concentrations were determined using a Pierce BCA Protein Assay Kit (Thermo Fisher Scientific). The proteins were separated on SDS–polyacrylamide gels and blotted onto polyvinylidene difluoride (PVDF) membranes (GE Healthcare, Chicago, IL, USA). The blots were blocked with 5% skim milk in Tris-buffered saline containing 0.1% Tween-20 (TBST), and then incubated overnight with the primary antibodies diluted in TBST containing 1% skim milk. The antibodies used in the study were: CHOP (Santa Cruz Biotechnology, Dallas, TX; sc-7351), ATF4 (GeneTex, Irvine, CA, USA; GTX101943), total PRDX4^14^, PRDX4t^9^, SPA17 (Proteintech, Rosemont, IL, USA; 13367-1-AP), GPX4 (Abcam, Cambridge, MA, USA; ab125066), PRDX1^23^, PRDX2 (AbFrontier, Seoul, Korea: LF-PA0091), PRDX3 (AbFrontier; LF-MA0044), PRDX6 (AbFrontier; LF-PA0011), SRX1 (Genetex; GTX51959), PRDX-SO_2/3_ (Abcam; ab16830), SOD1^24^, SOD2^24^, 4-HNE (JaICA Fukuroi, Japan; MHN-100P), and β-actin (GeneTex; GTX629630). After three washings with TBST, the blots were incubated with horseradish peroxidase (HRP)-conjugated anti-mouse (Santa Cruz Biotechnology; sc-2005) or anti-rabbit (Santa Cruz Biotechnology; sc-2004) secondary antibodies. After washing further, the bands were detected using an Immobilon western chemiluminescent HRP substrate (Merck Millipore, Burlington, MA, USA) on an image analyzer (ImageQuant LAS 500, GE Healthcare).

### In vitro fertilization (IVF)

IVF was performed as previously reported^25^ with minor modifications. Oocytes were collected from WT female mice with ICR backgrounds that had been superovulated by treatment with 7.5 IU each of pregnant mare serum gonadotropin (PMSG; ASKA Pharmaceutical, Tokyo, Japan) and human chorionic gonadotropin (hCG; ASKA Pharmaceutical). The cumulus cell-oocyte complex was inseminated with spermatozoa collected by squeezing from the cauda epididymis and placed into human tubal fluid medium (LifeGlobal Group, Guilford, CT, USA; ZHTF-100) supplemented with 4 mg/ml bovine serum albumin (BSA). Incubation was conducted at 37 °C in a humidified atmosphere of 5% CO_2_ in air.

### Testicular hyperthermia

Testicular hyperthermia was performed as described previously^26^. Briefly, testes were subjected to a hyperthermia treatment at 43 °C for 15 min by immersing the mice in a hot-water bath. After the heat treatment, mice were dried and returned to their cages. The mice were then sacrificed after 1 or 4 weeks, and the testes were collected for further analyses.

### RT-PCR

RNA from tissues was purified by means of ISOGEN II (Nippon Gene, Tokyo, Japan). cDNA was generated from 1 μg of total RNA per sample using a primescript cDNA synthesis kit (Takara Bio, Shiga, Japan). Semi-quantitative RT-PCR was performed using a Takara PCR Thermal Cycler Dice TP600 (Takara Bio) and AmpliTaq Gold DNA polymerase (Thermo Fisher Scientific) according to the manufacturer’s instructions. Amplification of the cDNAs using corresponding primers listed in Supplementary Table S3 followed by separation on a 2% agarose gel. The bands were detected by ethidium bromide staining on ImageQuant LAS 500.

### Statistical analysis

Statistical analyses were performed by Student’s *t*-test, one-way or two-way ANOVA followed by Tukey's test using the GraphPad Prism version 6.0 for Mac (GraphPad Software, San Diego, CA, USA; https://www.graphpad.com). A *P-*value of less than 0.05 was considered to be significant.

## Results

### PRDX4t is produced exclusively in the testis during spermatogenesis

**Figure 1:**
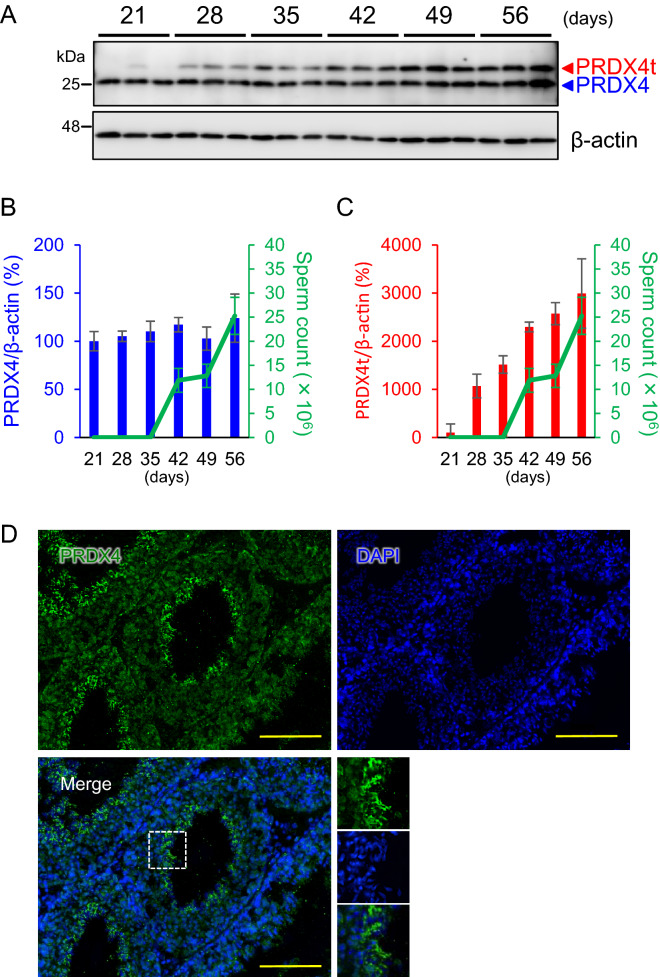
Age-dependent pattern of PRDX4t production in mice. **(A)** Western blotting of WT mouse testes using total PRDX4 and PRDX4t-specific antibodies. β-actin was used as an internal control. Testes at postnatal day 21, 28, 35, 42, 49, and 56 were analyzed (n = 3 per group). **(B)** Quantification of PRDX4 normalized to the corresponding β-actin. Data are the mean ± SEM. A line plot indicates changes in sperm counts (n = 4–5 per group). **(C)** Quantification of PRDX4t normalized to the corresponding β-actin. Data are the mean ± SEM. A line plot indicates changes in sperm counts (n = 4–5 per group). **(D)** The seminiferous tubules of the WT mouse were stained with a total PRDX4 antibody (green) and DAPI (blue). Scale bar, 100 μm.

To explore the potential role of PRDX4t in spermatogenesis and fertility, we first investigated PRDX4t protein production at different ages in mice. Western blot analyses indicated that the PRDX4t protein was produced in the testis at 28 days after birth (P28) and the levels were further increased in parallel with sexual maturation with an increase in sperm count whereas the conventional PRDX4 remained unchanged (Fig. 1A–C). The onset of PRDX4t production at P28 coincides with the elongation steps of spermatids during late spermatogenesis, indicating that the expression of the PRDX4t variant was induced during the first wave of spermatogenesis. In addition, RT-PCR analyses confirmed the age-dependent expression of PRDX4t in WT mouse testes (Supplementary Figure S1). Consistent with previous reports^12,13^, we confirmed that PRDX4t was produced exclusively in round spermatids, elongating spermatids, and spermatozoa in WT mouse testes by immunofluorescence analyses (Fig. 1D and Supplementary Figure S2), suggesting that it has a potential role in mouse spermatogenesis.

### PRDX4t-knockout male mice are fertile

**Figure 2:**
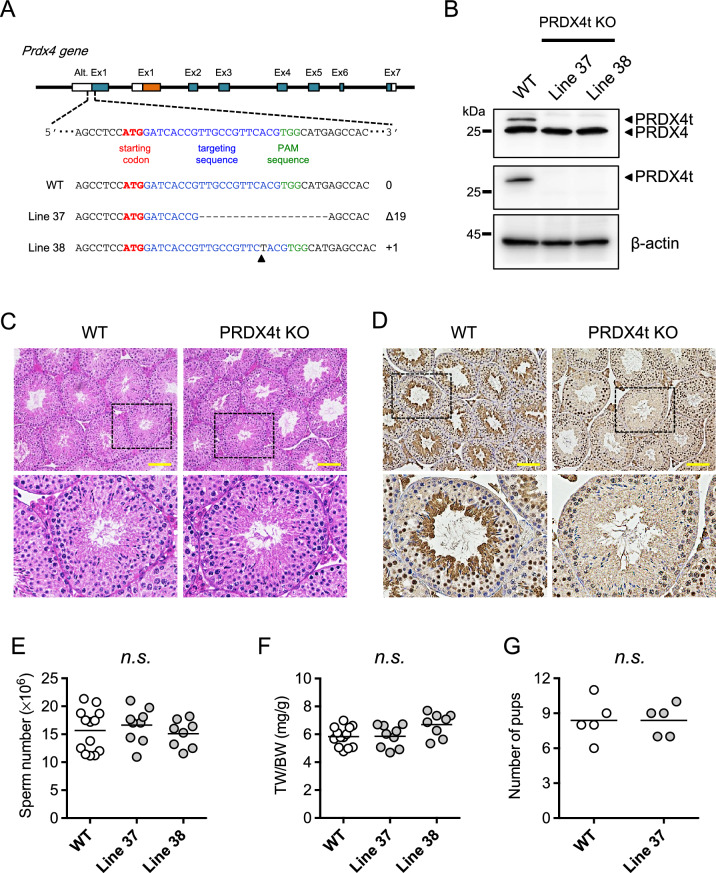
Establishing PRDX4t-deficient mice using CRISPR/Cas9 strategy. **(A)** Schematic representation of the genomic target sites in the Prdx4 gene. The alternative exon 1 (Alt. Ex 1) of the Prdx4 gene was targeted. Target sites are marked in blue, PAM sites are shown in green, and the vertical arrow represents an insertion. Dashed line represents deleted sequences. Male offspring harboring either a 19-bp deletion (Line 37) or a 1-bp insertion (Line 38) were used in this study. **(B)** Western blotting of testes using total PRDX4 or PRDX4t-specific antibodies. β-actin was used as a loading control. **(C)** H&E staining for testicular morphology. Representative images of testes from WT or PRDX4t KO (Line 37) mice at 12-weeks of age are shown. Scale bar, 100 µm. The square areas are enlarged and shown in the bottom panel. **(D)** Immunohistochemical analyses were performed using a PRDX4t-specific antibody. Representative images of testes from WT or PRDX4t KO (Line 37) mice at 12-weeks of age are shown. Scale bar, 100 µm. The square areas are enlarged and shown in the bottom panel. **(E)** Sperm counts of the mice of the indicated genotypes. Each dot represents an individual mouse (n = 8–13 per group). **(F)** Testis/body weight ratio of the mice of the indicated genotypes. Each dot represents an individual mouse (n = 8–13 per group). **(G)** Individual WT or PRDX4t KO male mouse was cohabitated with sexually mature WT female mice. Each dot represents the number of pups of each litter for WT or PRDX4t KO mice (n = 5 per group). Statistical analyses were performed using one-way ANOVA with Tukey's test **(E, F)**, or Student’s *t*-test **(G)**. *n.s.*, not significant. Bar, mean.

Because the *Prdx4t* gene is transcribed from the alternative exon 1 that is located upstream from the conventional promoter/exon 1, we designed sgRNAs that targeted locations near the translation start codon in the alternative exon 1 (Fig. 2A) and delivered them to mouse embryos by microinjection together with Cas9 protein. Mutations in the gene were screened by PCR, using primers spanning the target sites and ultimately confirmed by Sanger sequence analyses. For further study, we chose two mouse lines harboring either a 19 bp deletion or a 1 bp insertion mutation (line 37 and 38, respectively). We then detected the PRDX4t protein in the testes of sexually matured male mice by western blotting. The results showed that neither of the lines produced PRDX4t but robustly produced conventional PRDX4 (Fig. 2B). Hematoxylin and eosin (H&E) staining of testes revealed that the KO mouse seminiferous tubules had a normal architecture and that the distribution of germ cells was well organized (Fig. 2C). Immunohistochemistry examination using a PRDX4t-specific antibody revealed that the production of PRDX4t was completely abolished in the KO mouse testes (Fig. 2D). We then analyzed the phenotype of the PRDX4t KO mice with a particular focus on spermatogenesis. The findings showed that the sperm counts of PRDX4t KO mice were nearly the same as WT mice (Fig. 2E). Furthermore, the testis/body weight ratios of the PRDX4t KO mice and WT mice were essentially the same (Fig. 2F). To determine the fertility of the PRDX4t KO males, we performed a fecundity test using PRDX4t KO males bred with fertility-proven adult WT females. Our breeding data showed that the litter sizes from the PRDX4t KO males were similar to those produced by WT mating pairs (Fig. 2G). These collective results indicate that mice that are deficient in PRDX4t, even though it is exclusively produced in WT mouse testes, were fully fertile.

### PRDX4 and PRDX4t double-deficient male mice are fertile

**Figure 3:**
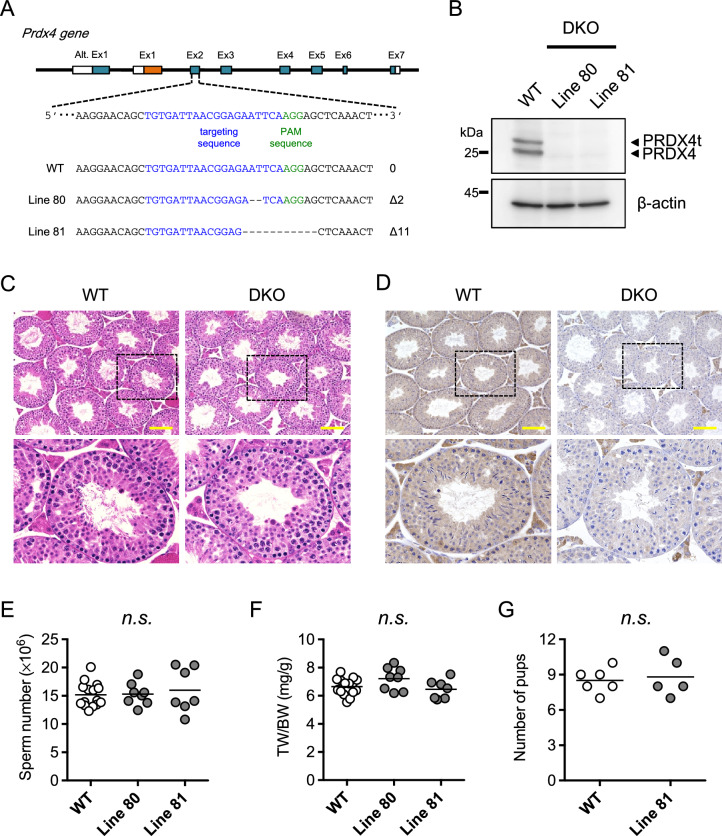
Establishing PRDX4/PRDX4t DKO mice using a CRISPR/Cas9 strategy. **(A)** Schematic representation of the genomic target sites in the Prdx4 gene. Exon 2 of the Prdx4 gene was targeted. Target sites and PAM sites are marked in blue and green, respectively. Dashed line represents deleted sequences. The male offspring harboring either a 2-bp deletion (Line 80) or a 11-bp deletion (Line 81) was used for this study. **(B)** Western blotting of testes using a total PRDX4 antibody. β-actin was used as a loading control. **(C)** H&E staining for testicular morphology. Representative images of testes from WT or DKO (Line 81) mice at 12-weeks of age are shown. Scale bar, 100 µm. The square areas are enlarged and shown in the bottom panel. **(D)** Immunohistochemical analyses were performed using a total PRDX4 antibody. Representative images of testes from WT or DKO (Line 81) mice at 12-weeks of age are shown. Scale bar, 100 µm. The square areas are enlarged and shown in the bottom panel. **(E)** Sperm counts of the mice of the indicated genotypes. Each dot represents an individual mouse (n = 7–15 per group). **(F)** Testis/body weight ratio of the mice of the indicated genotypes. Each dot represents an individual mouse (n = 7–15 per group). **(G)** Individual WT or DKO male mice were cohabitated with sexually mature WT female mice. Each dot represents the number of pups of each litter for WT or DKO mice (n = 5–6 per group). Statistical analyses were performed using one-way ANOVA with Tukey's test (E and F), or Student’s *t*-test **(G)**; *n.s.*, not significant. Bar, mean.

Since the conventional PRDX4 is also produced in the testes at high levels and may compensate for PRDX4t function, we attempted to disrupt the expression of both PRDX4 and PRDX4t in mice by means of the CRISPR/Cas9 system. We targeted the exon 2 of *Prdx4* gene, which is common to both PRDX4 and PRDX4t and essential for enzymatic reactions (Fig. 3A). Sequence analyses revealed that the mutant lines harbored either a 2 or 11 bp deletion mutation (line 80 and 81, respectively). Neither the PRDX4 protein nor the PRDX4t protein (Fig. 3B) was detected in both lines, indicating that these animals were doubly deficient in PRDX4 and PRDX4t expression (hereafter referred to as DKO mice). Both male and female DKO mice were viable and displayed no developmental abnormalities compared with WT littermates.

**Figure 4:**
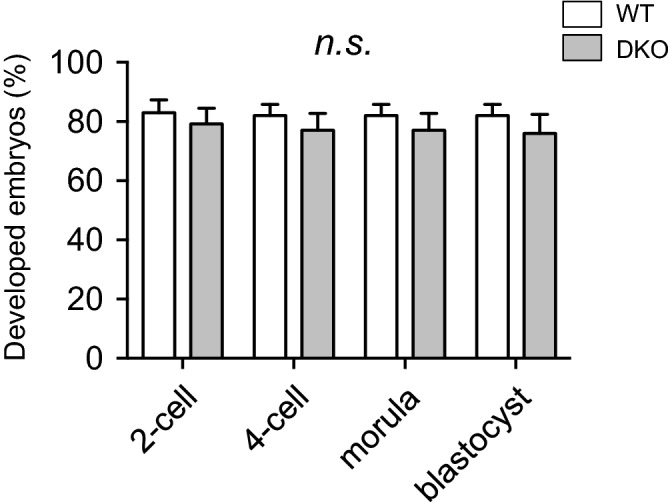
Fertilizing ability of mouse spermatozoa. IVF data show the rate (%) of developing embryos using spermatozoa from the indicated male mice. Oocytes were collected from WT females and inseminated with spermatozoa from WT or DKO (Line 81) mice (n = 4 per group). Data are the mean ± SEM. Statistical analyses were performed using two-way ANOVA with Tukey’s test. There were no significant differences between the two groups (*n.s.*, not significant).

H&E staining of testicular sections from 12-week-old mice showed that there was no structural abnormality in the testes between these genotypic mice (Fig. 3C), although immunohistochemistry revealed that the production of both PRDX4 and PRDX4t were completely abolished in the DKO mouse testes (Fig. 3D). No significant differences were observed in sperm counts (Fig. 3E) or testis-to-body weight ratios (Fig. 3F). Furthermore, the numbers of pups per litter produced by mating DKO males with WT females were similar to those produced by WT mating pairs (Fig. 3G). We next examined the function of spermatozoa that swam up from the dissected cauda epididymis. In vitro-fertilization tests of the spermatozoa from both WT and DKO mice using oocytes from WT mice again showed no significant difference in fertilizing ability (Fig. 4). Overall, these results indicate that the disruption of both PRDX4 and PRDX4t has no obvious effects on either male fertility or sperm function under normal laboratory conditions.

### Heat treatment induces testicular damages and increases ER stress to the same extent in testes of DKO and WT mice

**Figure 5:**
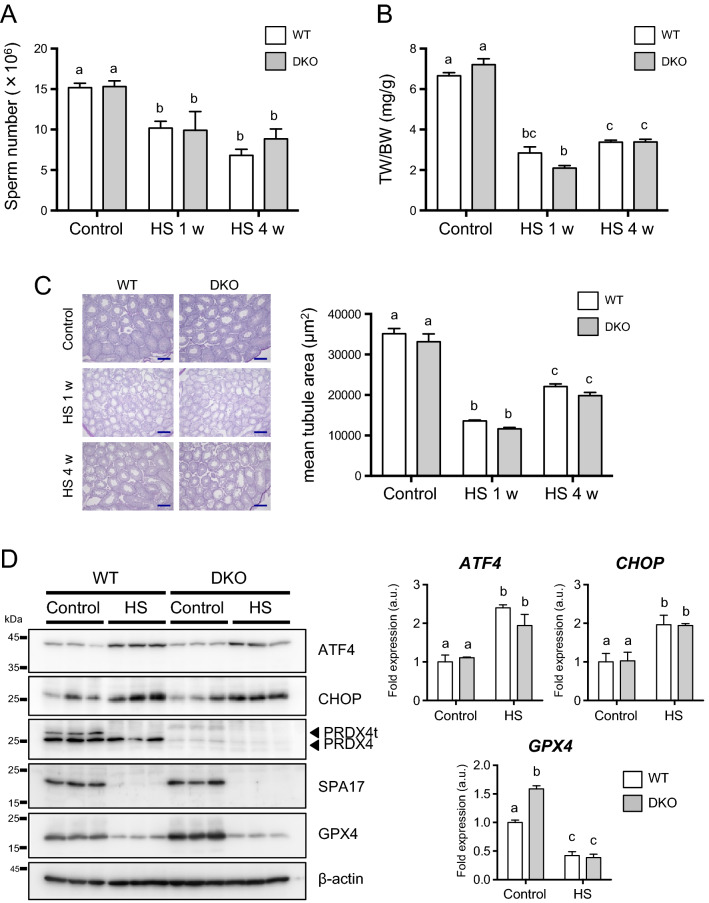
Morphological changes and ER stress responses after testicular hyperthermia in mice. **(A)** WT and DKO (Line 80) mice were exposed to testicular hyperthermia (HS). After 1 or 4 weeks, the sperm counts of the mice in the indicated genotype were determined (n = 3 for each group). Data are the mean ± SEM. Statistical analyses were performed using two-way ANOVA with Tukey's test. Different letters indicate statistically significant differences (*p* < 0.05). **(B)** WT and DKO (Line 80) mice were exposed to testicular hyperthermia (HS). After 1 or 4 weeks, the testis/body weight ratio of the mice of the indicated genotype was determined (n = 3 for each group). Data are the mean ± SEM. Statistical analyses were performed using two-way ANOVA with Tukey's test. Different letters indicate statistically significant differences (*p* < 0.05). **(C)** WT and DKO (Line 80) mice were exposed to testicular hyperthermia (HS). After 1 or 4 weeks, PAS staining for testicular morphology for the indicated genotype are shown. The sections were counterstained with Mayer's hematoxylin. Scale bar, 200 μm. The right panel depicts the quantification of mean tubule area (n = 4 for each group). Data are the mean ± SEM. Statistical analyses were performed using two-way ANOVA with Tukey's test. Different letters indicate statistically significant differences (*p* < 0.05). **(D)** WT and DKO (Line 80) mice were exposed to testicular hyperthermia (HS). After 1 week, testes were collected and the protein production levels of ATF4, CHOP, PRDX4, PRDX4t, SPA17, GPX4, and β-actin in testes were determined by western blotting (n = 3 for each group). The graph depicts the quantification of ATF4, CHOP, and GPX4 normalized to the corresponding β-actin. Data are the mean ± SEM. Statistical analyses were performed using two-way ANOVA with Tukey's test. Different letters indicate statistically significant differences (*p* < 0.05).

Because spermatogenic cells are vulnerable to heat stress^23,27^, we determined the sensitivity of both DKO and WT mice to a hyperthermia treatment. We observed a decline in sperm counts and the testis-to-body weight ratio in both genotypic mice after the heat treatment, but the reduction was not statistically different between the two genotypic mice (Fig. 5A,B). There were also declines in the mean diameters of seminiferous tubules of both the WT and DKO mouse groups after the heat treatment compared with the corresponding control group, but they were not significantly different between the two genotypic mice (Fig. 5C).

We next examined the issue of whether testicular hyperthermia alters the production of canonical marker proteins for ER stress, including activating transcription factor 4 (ATF4) and the C/EBP homology protein (CHOP), in testes by western blotting. The heat treatment resulted in an increase in the levels of both the ATF4 and CHOP protein in testes of both genotypic mice, but there was also no significant difference between the two genotypic mice groups (Fig. 5D). Overall, these results indicate that heat treatment induced testicular damage and increased ER stress in testes of both mice to the same extent. On the contrary, a marked reduction in PRDX4t as well as the sperm surface protein Sp17 (SPA17) was observed in testes in both genotypic mice after heat treatment, but there was, again, no difference between the genotypic groups (Fig. 5D). Phospholipid hydroperoxide glutathione peroxidase encoded by GPX4 specifically detoxifies phospholipid hydroperoxides, and plays a most important role in the testis^20^. Of note, the levels of GPX4 were much higher in the DKO mouse testes under non-treatment condition, which decreased after testicular heating.

### GPX4 is present in higher levels in the testis as well as caput epididymis of the DKO mice

**Figure 6:**
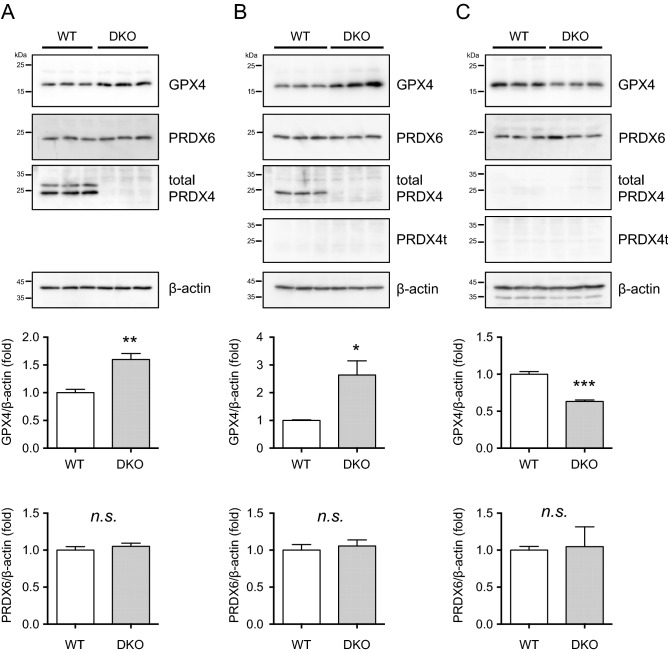
The differential GPX4 production levels in the testis and epididymis. Western blotting of **(A)** testis, **(B)** caput epididymis, and **(C)** cauda epididymis tissues collected from WT or DKO (Line 81) mice using GPX4 or PRDX6 antibodies. β-actin was used as a loading control. The graph depicts the quantification of each protein normalized to the corresponding β-actin. Data are the mean ± SEM (n = 3 for each group). Statistical analyses were performed using Student’s *t*-test. *p < 0.05; **p < 0.01; ***p < 0.001. *n.s.*, not significant.

Because GPX4 is reportedly essential for functional sperm formation^28^, and the GPX4 protein levels are elevated in the DKO mouse testes, we examined the protein levels of GPX4 as well as PRDX4 and PRDX4t in the testis and epididymis of these mice. The GPX4 protein was detected in all three tissues. It should be noted that the levels of the GPX4 protein were much higher in the testis (Fig. 6A) and caput epididymis (Fig. 6B) of the DKO mice compared with the corresponding values for the WT mice, but this was not the case for the cauda epididymis (Fig. 6C). We also examined the protein level of GPX4 in PRDX4t KO mice, but there was no difference between WT and PRDX4t KO mice (Supplementary Figure S3).

Because PRDX6 has also a prime role in male fertility^29–32^, we examined the protein levels of PRDX6 in the testis and epididymis of these mice. However, there were no significant changes between WT and DKO mice (Fig. 6A–C). We next examined the protein levels of major enzymes involved in redox signaling in testes by western blotting. The protein levels of PRDX1, PRDX2, PRDX3, sulfiredoxin (SRX1), superoxide dismutase 1 (SOD1), and SOD2 were not significantly different between the two groups (Supplementary Figure S4). Also, the levels of hyperoxidized PRDX (PRDX-SO_2/3_) and 4-hydroxynonenal (4-HNE; the product of lipid peroxidation) did not change significantly between the two groups. The epididymal histology suggested no overt differences between these genotypes (Supplementary Figure S5). While both PRDX4 and PRDX4t proteins were present in the testis, only PRDX4 was detected in caput epididymis and none was detected in the cauda epididymis.

## Discussion

To gain additional insight into the roles of PRDX4t in spermatogenesis, we generated mice that do not express the PRDX4t protein using a genome-editing technique and performed analyses on the resulted male mice with respect to male fecundity. We first made genetically modified mice that were selectively lacking in producing the PRDX4t protein but produced conventional PRDX4 normally (Fig. 2). As a result, we found no aberrant phenotype in either spermatogenesis or fertilizing ability in the PRDX4t-deficient male mice compared with the WT mice. To eliminate possible compensation for the depleted PRDX4t expression by conventional PRDX4, we then prepared double deficient mice that did not produce either PRDX4 or PRDX4t. Again, no phenotypic abnormality was observed in the fertilizing ability of sexually mature male mice or the production and function of spermatozoa (Figs. 3 and 4). Thus far the results indicate that, despite the testis-specific expression, PRDX4t is not essential for spermatogenesis or fecundity in mice. Analyses of the testis and epididymis of these mice, however, showed that the production of GPX4 is elevated in both the testis and caput epididymis (Figs. 5 and 6), suggesting compensation for the absence of PRDX4 and PRDX4t by GPX4 and possibly other antioxidant enzymes (e.g. PRDXs) as well. Although we could not detect any changes in PRDX6 levels in the testis and epididymis, there was an increase in the amount of PRDX6 in in the epididymis from rats challenged with the in vivo oxidative stress^32,33^, possibly through a mechanism involving epididymosomes^34^. Thus, it is possible that this mechanism is occurring in the epididymis of the DKO mice to protect epididymal spermatozoa from oxidative stress to avoid impairment of fertilizing ability.

Extensive attempts using genomic and transcriptomic approaches have been made to determine the function of the genes that are predominantly expressed in the testis and these efforts currently include over 2300 genes^35^. Since then, 54 genes that are evolutionarily conserved and show testis-enriched expression have been found to be not essential for male fertility in individual cases of the gene in the mouse^36^. Recent studies based on CRISPR/Cas9-mediated genome-editing strategies further confirmed that such genes are essentially not required for male fertility in the mouse^37–39^. Considering these facts, functional redundancy represents the likely explanation for the lack of obvious phenotypic abnormalities in the PRDX4t KO mice.

Because disulfide bond formation in oxidative protein folding is a unique function of PRDX4 in the ER^5,6^ and because PRDX4t exerts its peroxidase activity in the same manner as PRDX4^16^, it is conceivable that PRDX4t also catalyzes disulfide bond formation in spermatogenic cells by consuming hydrogen peroxide. The disulfide bond formation occurs on sulfhydryl groups in sperm protamines, which proceeds only partially in the testis and is largely occurs in the epididymis where sperm cells undergo maturation. Although there appeared to be discrepancy in the production of GPX4 and PRDX4 proteins in mouse tissues (Figs. 5 and 6), the production of abundant levels of PRDX4t in the testis but not in the epididymis implies that PRDX4t, together with GPX4, contribute to the preliminary disulfide bond formation that occurs in the seminiferous tubules of testis. Accordingly, the up-regulation of GPX4 in the testis and caput epididymis of the DKO mice could be due to a compensatory response under conditions of a PRDX4t deficiency.

GPX4 is present in high levels in the epididymis as well as the testis. Three GPX4 variants, mitochondrial, cytosolic and nucleolar GPX4, are abundantly transcribed from one gene by alternative splicing^41,42^. While the depletion of all GPX4 isoforms causes early embryonic lethality in mice^41,43^, mice lacking mitochondrial GPX4 are viable but the males are infertile with severe structural abnormalities in the sperm midpiece that is constructed mainly by mitochondria^44^. Higher protein thiol contents in the spermatozoa of the mitochondrial GPX4-deficient mice compared with WT spermatozoa provide support for mitochondrial GPX4 functioning in the disulfide bond formation. The spermatocyte-specific knockout of GPX4 consistently causes male infertility in mice, and the isolated spermatozoa are unable to fertilize oocytes in vitro^45^. The results observed for the genetically modified mice may be a mimetic of oligoasthenozoospermia found in sterile male with a GPX4 insufficiency^46^. However, mice that are deficient in the nuclear form of GPX4 are viable and fully fertile, even though the spermatozoa are more prone to decondensation compared with those from WT mice during epididymal maturation^28,40,47^. Thus, GPX4 appears to have an indispensable role in spermatogenesis universally. Considering the function of PRDX4 in the rapid and non-specific thiol oxidase in the ER^7^, PRDX4t may have a role in the initial compaction of protamines in the early stages of the spermatogenic process.

Testes are rich in polyunsaturated fatty acids, and the machineries for spermatogenesis are prone to oxidative modification and dysfunction. A deficiency of PRDX4/PRDX4t, therefore, would be expected to result in increased levels of hydrogen peroxide, which would then lead to sensitive molecules being oxidatively damaged. Such sensitive molecules would include thiols and polyunsaturated fatty acids and the production of lipid peroxides^14^. Uncontrolled formation of supporting disulfide bonds caused by elevated levels of hydrogen peroxide would lead to structurally abnormal chromatin and result in abnormal spermatogenesis^20^. Thus, it is also plausible that increased levels of GPX4 could compensate for the decreased antioxidant capacity caused by the depletion of PRDX4t and support spermatogenesis.

In conclusion, our study shows, for the first time, that the absence of PRDX4t has no apparent influence on the process of spermatogenesis, even though it is specifically expressed in the testis. Although there was the up-regulation of GPX4 in the testis and the caput epididymis under PRDX4/PRDX4t deficiency, we presume that other unidentified factors are also involved. Future studies are necessary to investigate the exact mechanisms that act to compensate for the disrupted PRDX4t function.

## Supplementary information

Supplementary Information 1.

Supplementary Information 2.

## Abbreviations

ATF4: Activating transcription factor 4
CHOP: C/EBP homology protein
DKO: Double knockout
ER: Endoplasmic reticulum
ERO1: Endoplasmic reticulum oxidoreductin 1
GPX4: Glutathione peroxidase 4
IVF: In vitro fertilization
KO: Knockout
PRDX4: Peroxiredoxin 4
PRDX4t: Testis-specific peroxiredoxin 4
PDI: Protein disulfide isomerase
ROS: Reactive oxygen species
