# Supplementary material for: Testis-specific peroxiredoxin 4 variant is not absolutely required for spermatogenesis and fertility in mice
Source: Sci Rep. 2020 Oct 21;10:17934. doi: 10.1038/s41598-020-74667-9 (PMC7577974; doi:10.1038/s41598-020-74667-9)
Supplement: Supplementary file 1 — Supplementary Information 1. [file 41598_2020_74667_MOESM1_ESM.pdf]

Supplementary information

Testis-specific peroxiredoxin 4 variant is not absolutely required for spermatogenesis and fertility in mice

Takujiro Homma[1], Toshihiro Kurahashi[1,a], Naoki Ishii[1], Nobuyuki Shirasawa[2], and Junichi Fujii[1]

[1]Department of Biochemistry and Molecular Biology, Graduate School of Medical Science, Yamagata University

[a]Present address: Department of Cellular Regenerative Medicine, Graduate School of Medical Science, Kyoto Prefectural University of Medicine

[2]Department of Rehabilitation, Faculty of Medical Science and Welfare, Tohoku Bunka Gakuen University, Sendai 981-8551, Japan.

Supplementary data: 15 Supplementary Figures and 3 Supplementary Tables

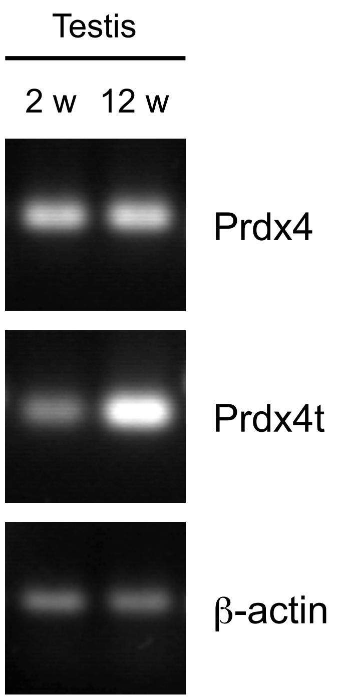

Supplementary Figure S1. RT-PCR analyses of Prdx4t gene expression in the testis at different ages. Total RNA was isolated from testes of WT mice at 2 or 12 weeks of age and analyzed in RT-PCR with primers listed in Supplementary Table S3. β-actin was used as a loading control.

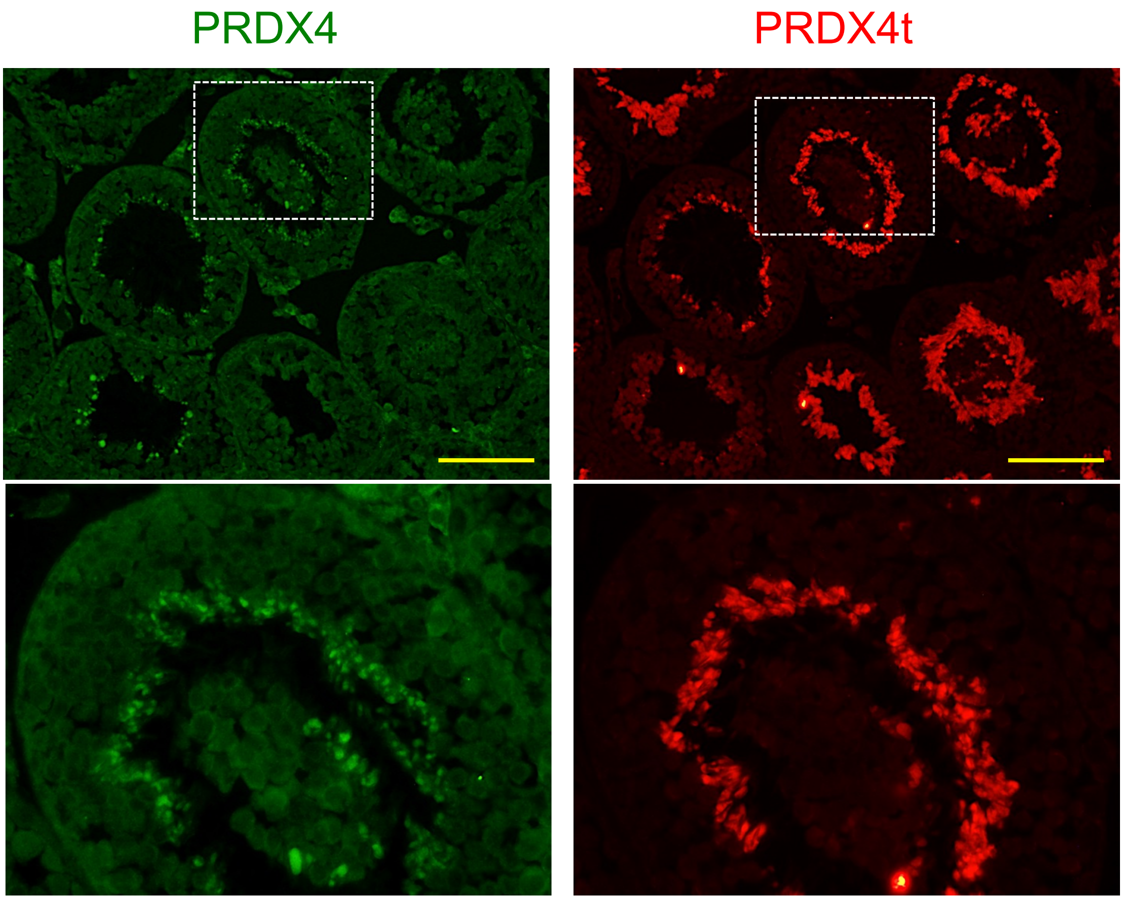

Supplementary Figure S2. The immunostaining was performed in adult WT mouse testes using total PRDX4 (green) or PRDX4t-specific (red) antibodies. The square areas are enlarged and shown in the bottom panel. Scale bar, 100 μm.

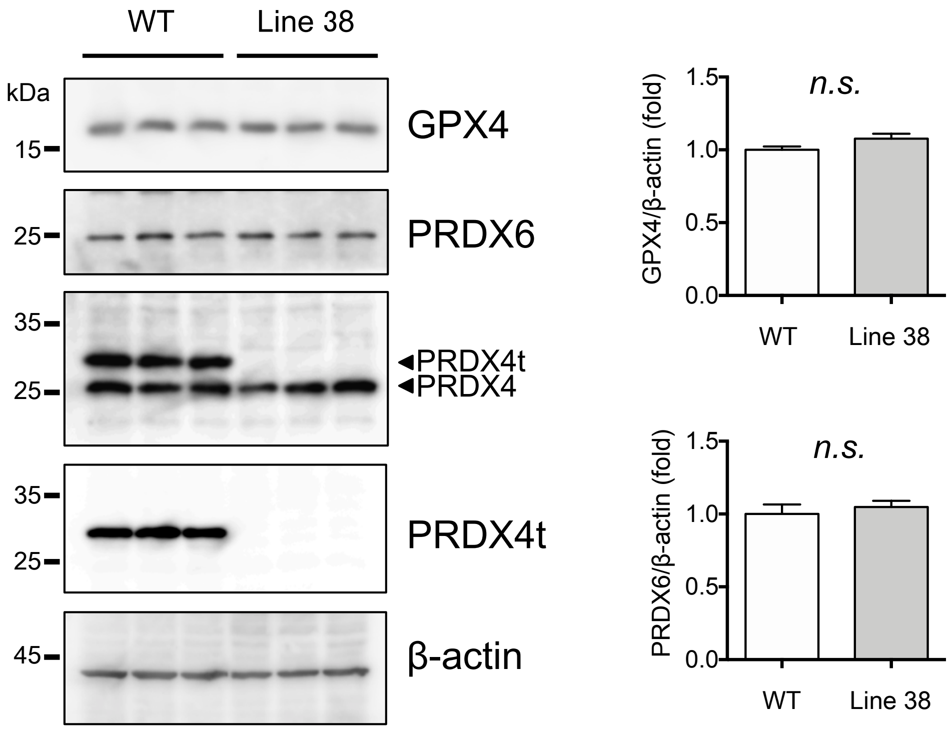

Supplementary Figure S3. Western blotting of testes collected from WT or PRDX4t KO (Line 38) mice using GPX4 or PRDX6 antibodies. β-actin was used as a loading control. The graph depicts the quantification of each protein normalized to the corresponding β-actin. Data are the mean ± SEM (n=3 for each group). Statistical analyses were performed using Student's *t*-test. *n.s.*, not significant.

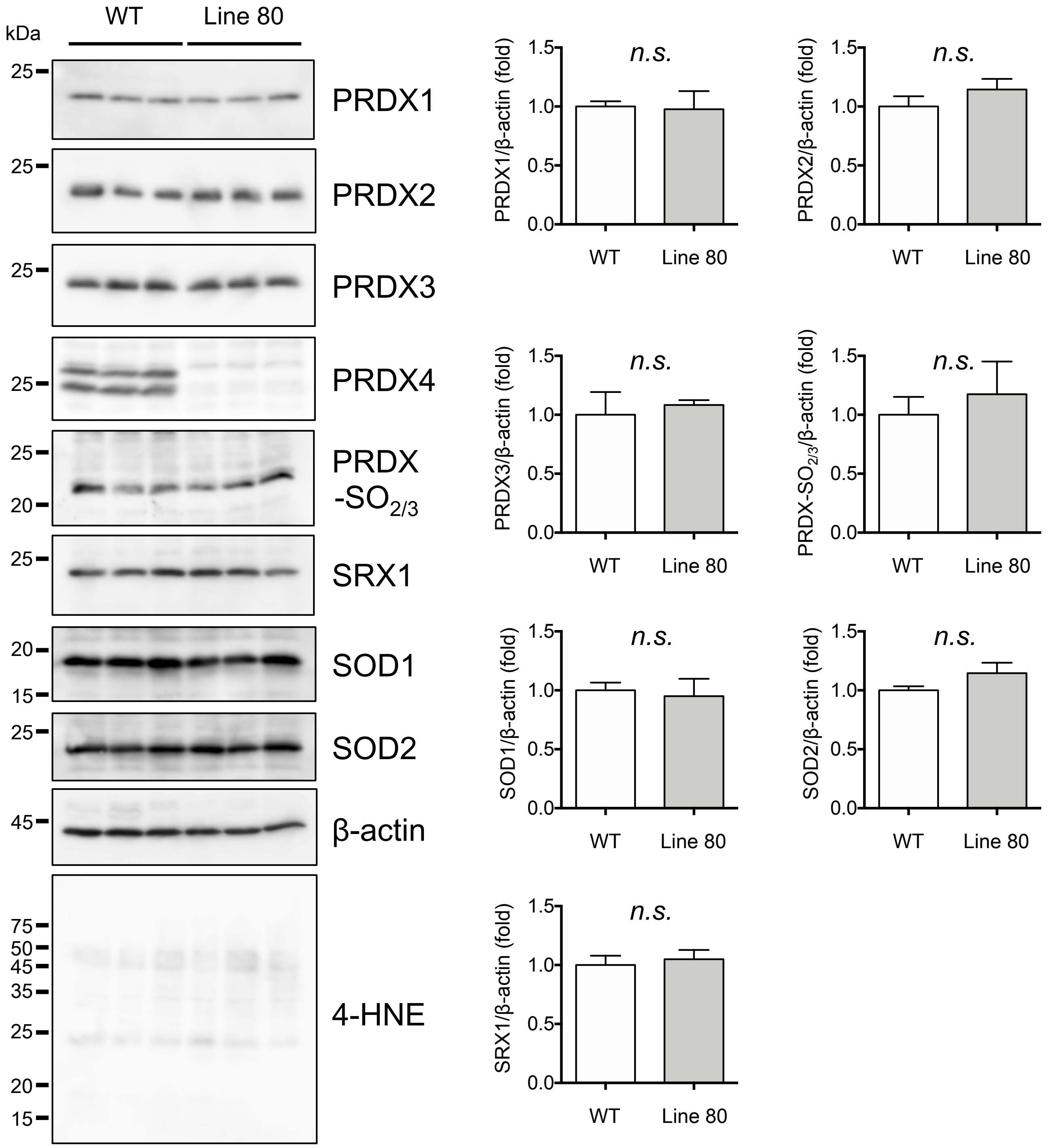

Supplementary Figure S4. Western blotting of testes collected from WT or DKO (Line 80) mice using PRDX1, PRDX2, PRDX3, PRDX4, PRDX-$SO_{2/3}$, SRX1, SOD1, SOD2, or 4-HNE antibodies. β-actin was used as a loading control. The graph depicts the quantification of each protein normalized to the corresponding β-actin. Data are the mean ± SEM (n=3 for each group). Statistical analyses were performed using Student's *t*-test. *n.s.*, not significant.

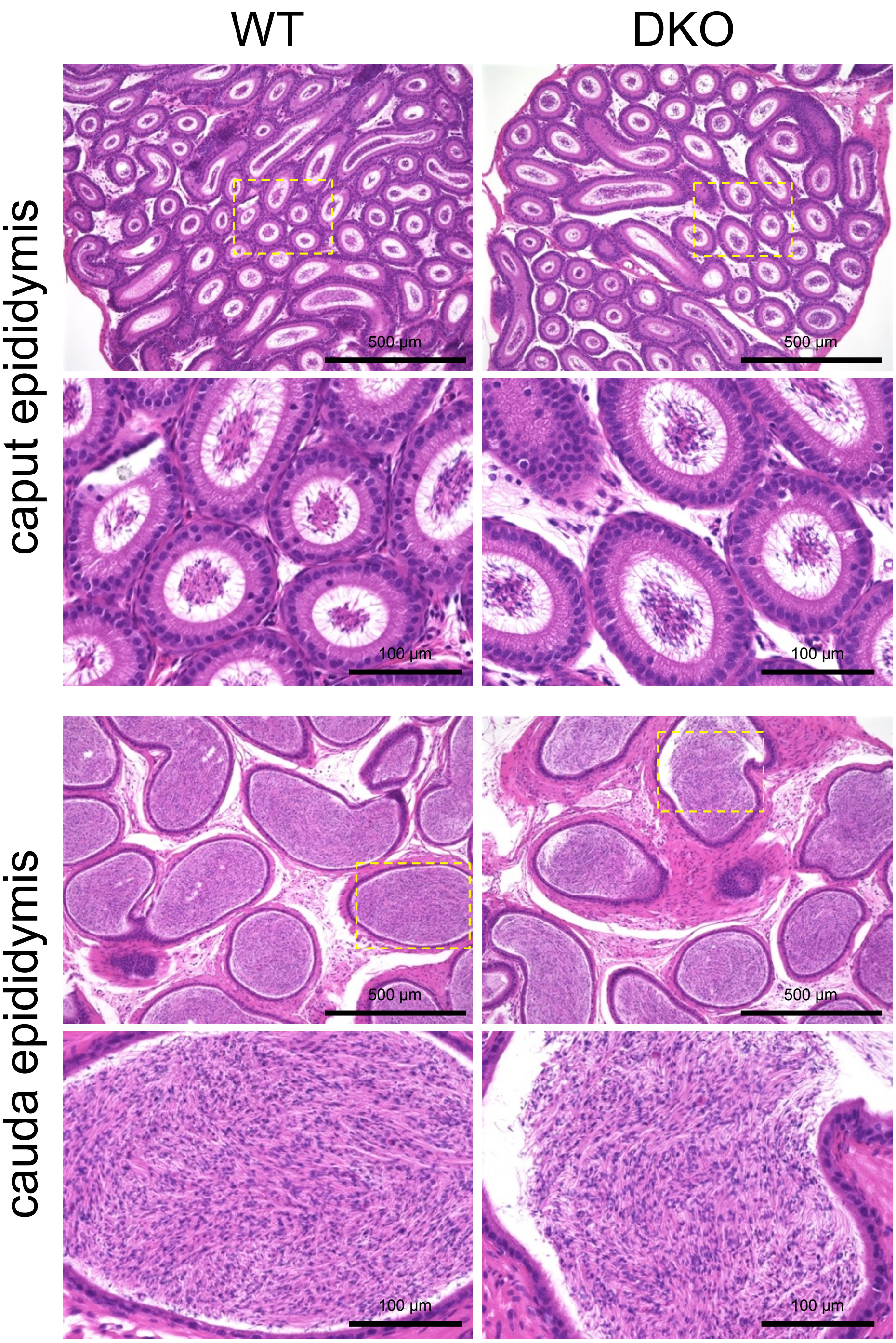

Supplementary Figure S5. Histological comparison of the epididymis.
Representative images of H&E staining of caput and cauda epididymis from WT and DKO (Line 81) mice at 12-weeks of age are shown. The square areas are enlarged and shown in the bottom panel.

Figure 1A: total PRDX4

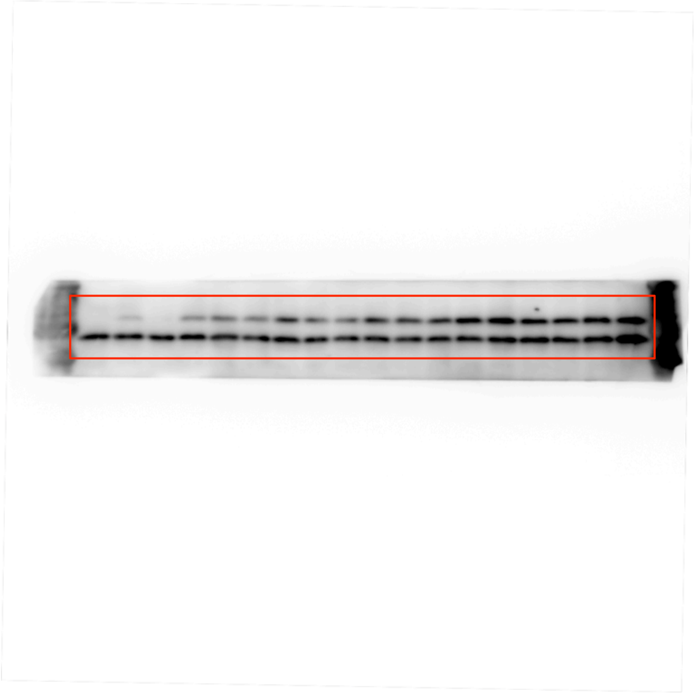

Figure 1A: β-actin

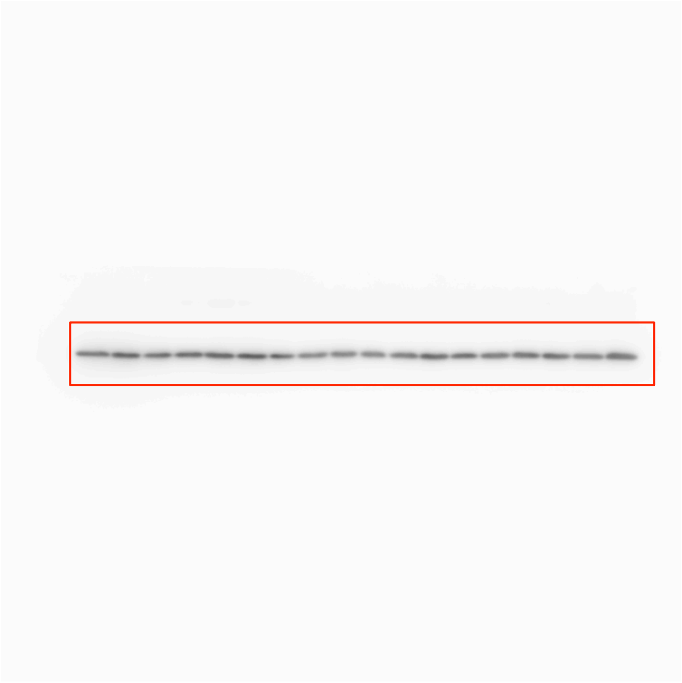

Supplementary Figure S6. Uncropped images of western blot presented in Figure 1A.

Figure 2B: total PRDX4

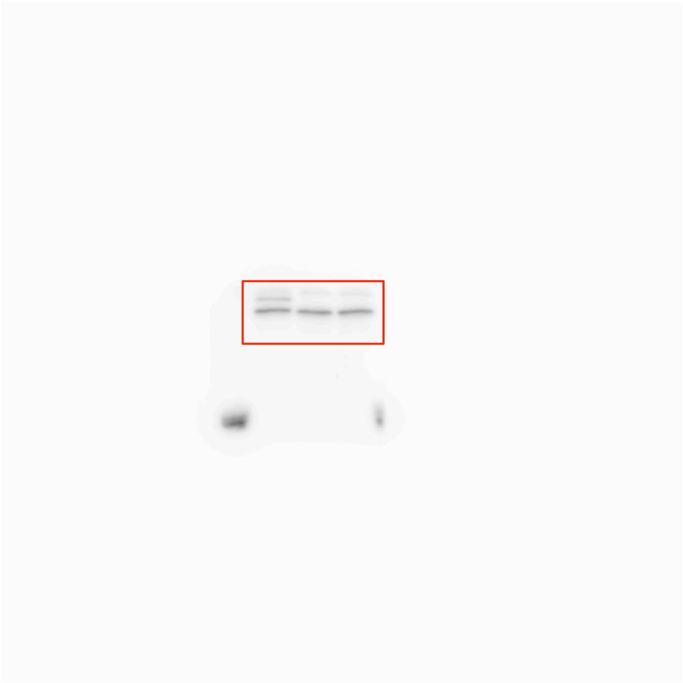

Figure 2B: PRDX4t specific

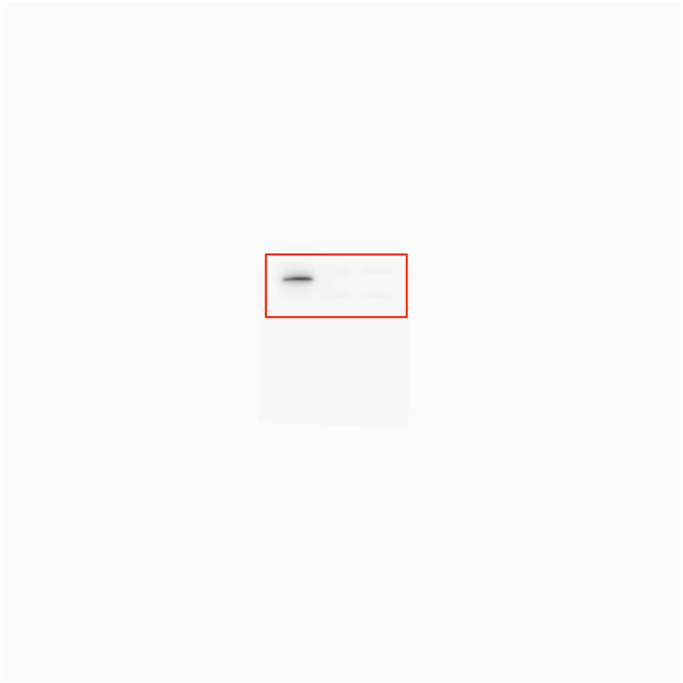

Figure 2B: β-actin

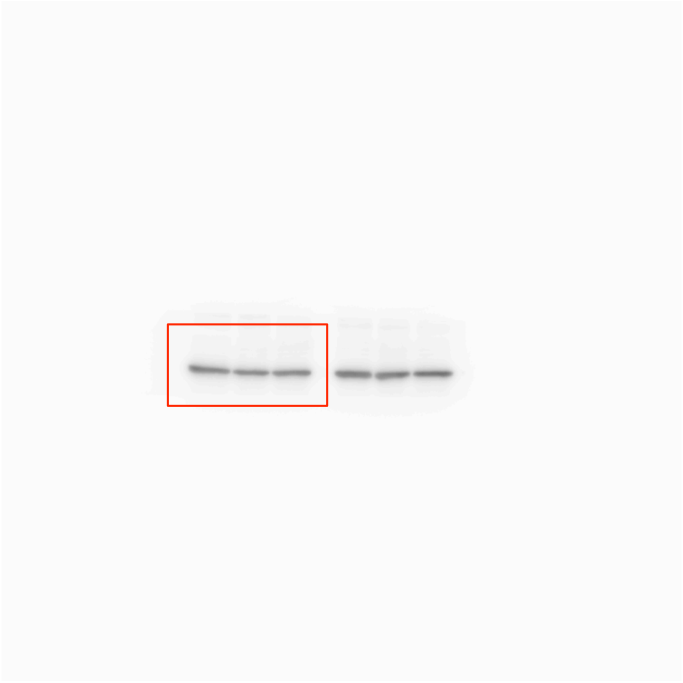

Supplementary Figure S7. Uncropped images of western blot presented in Figure 2B.

Figure 3B: total PRDX4

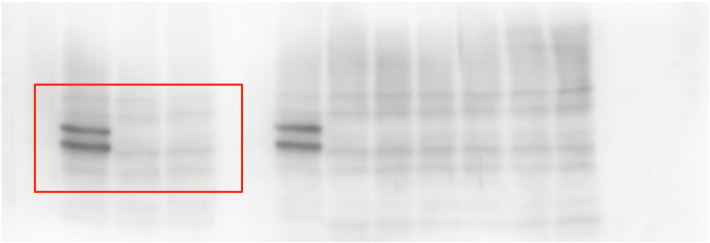

Figure 3B: β-actin

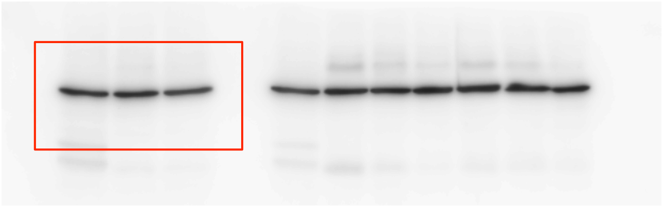

Supplementary Figure S8. Uncropped images of western blot presented in Figure 3B.

Figure 5D: ATF4

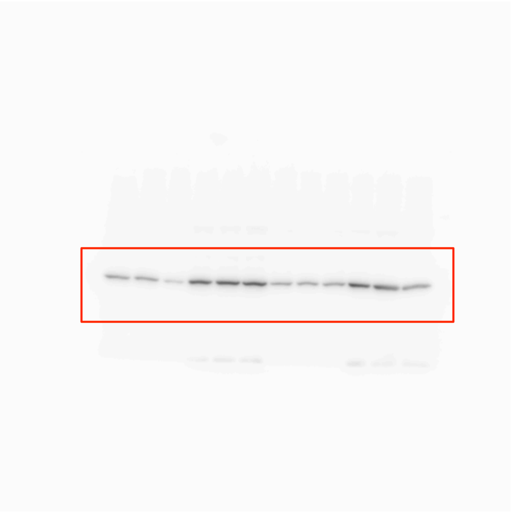

Figure 5D: CHOP

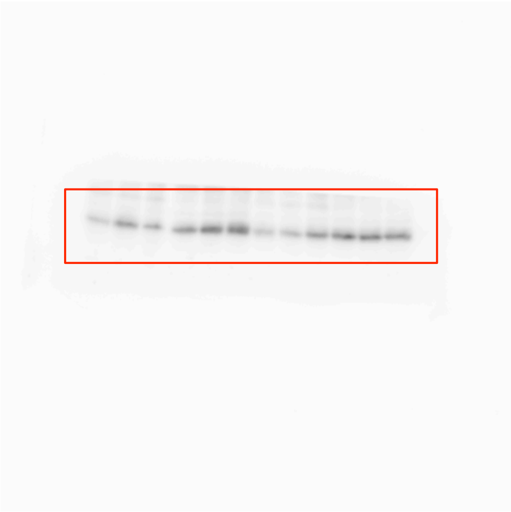

Figure 5D: total PRDX4

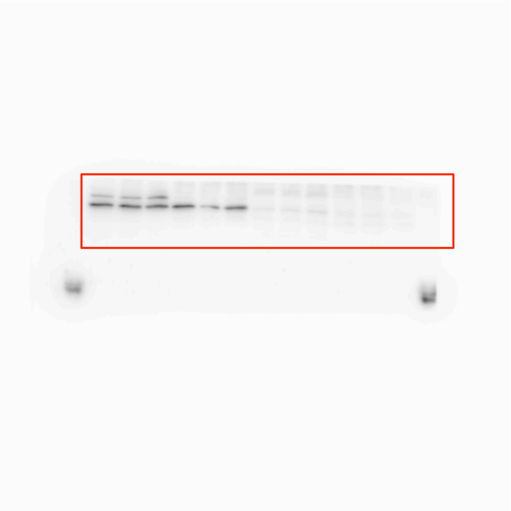

Figure 5D: SPA17

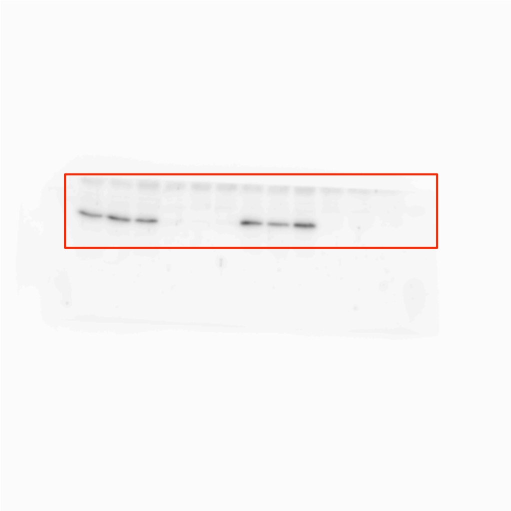

Figure 5D: GPX4

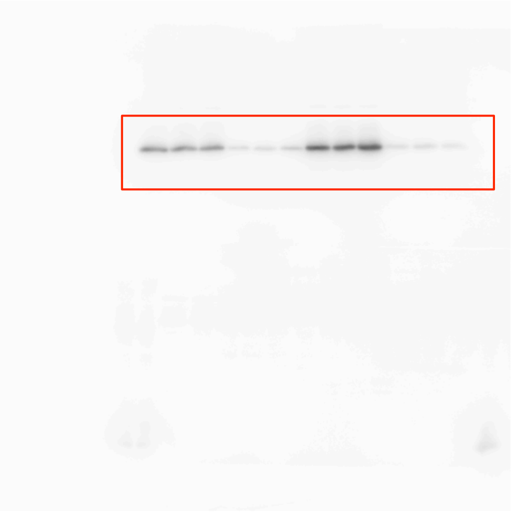

Figure 5D: β-actin

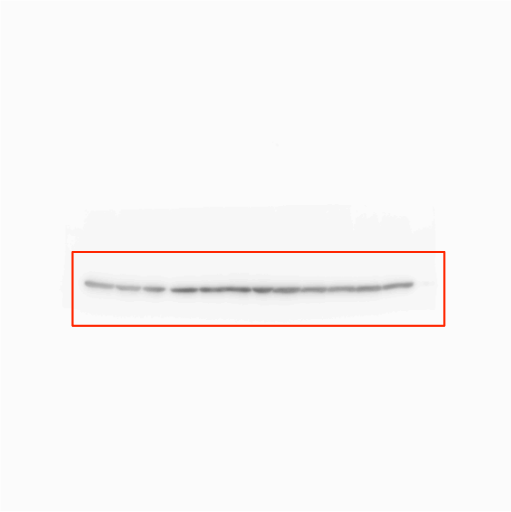

Supplementary Figure S9. Uncropped images of western blot presented in Figure 5D.

Figure 6A: GPX4

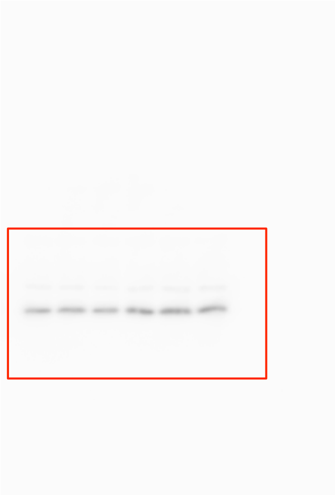

Figure 6A: PRDX6

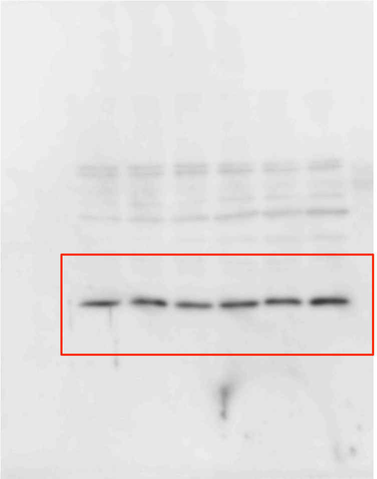

Figure 6A: total PRDX4

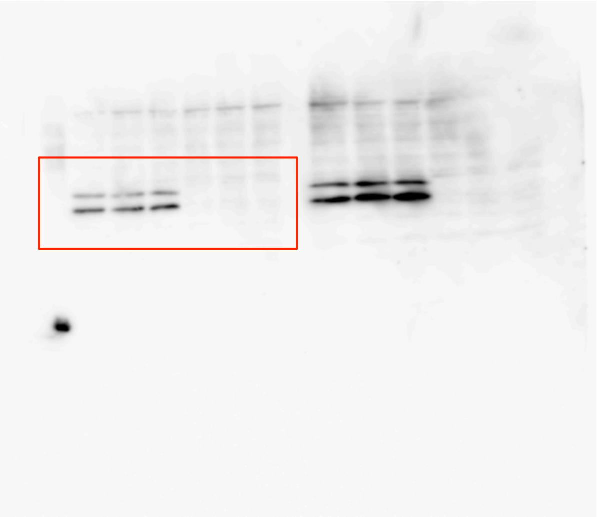

Figure 6A: β-actin

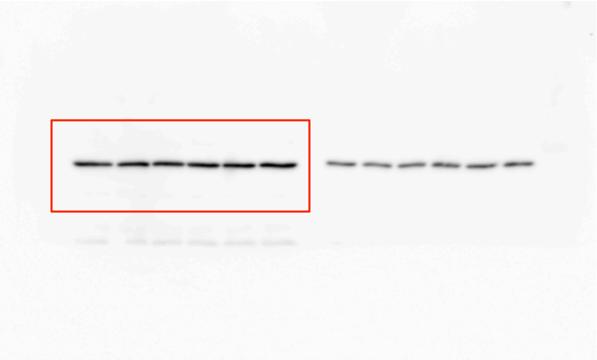

Supplementary Figure S10. Uncropped images of western blot presented in Figure 6A.

Figure 6B: GPX4

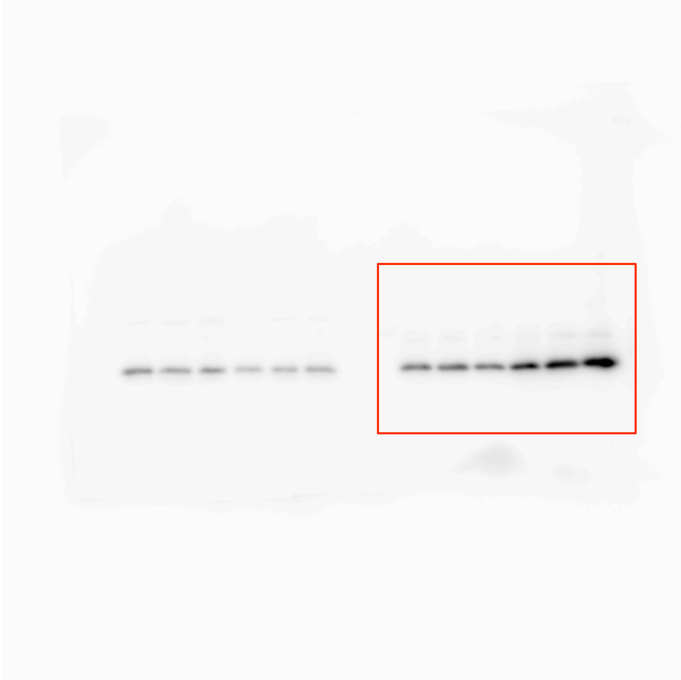

Figure 6B: PRDX6

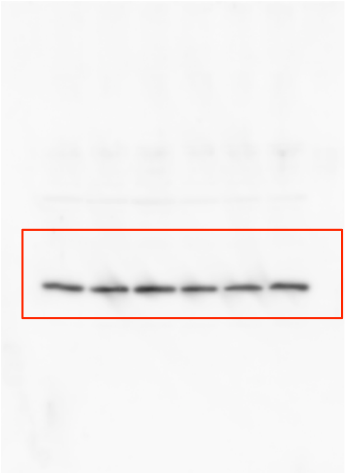

Figure 6B:
total PRDX4 (top) and PRDX4t specific (bottom)

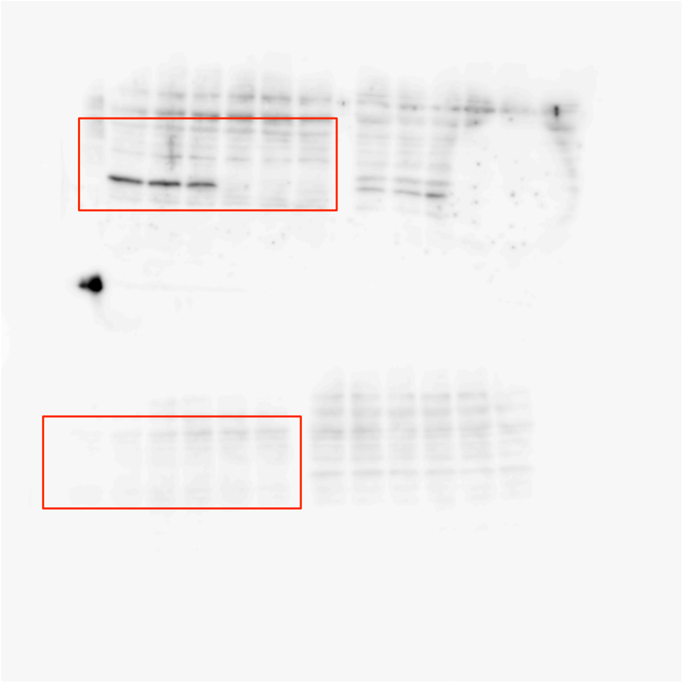

Figure 6B: β-actin

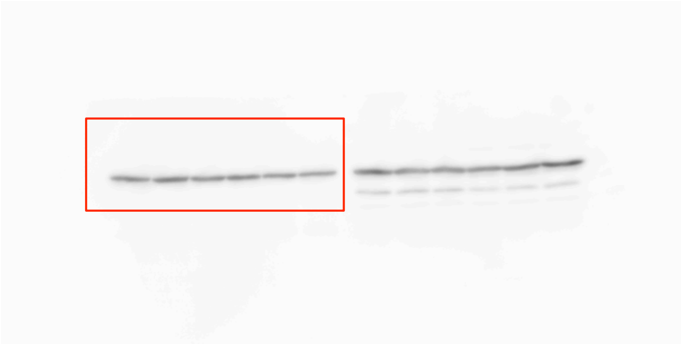

Supplementary Figure S11. Uncropped images of western blot presented in Figure 6B.

Figure 6C: GPX4

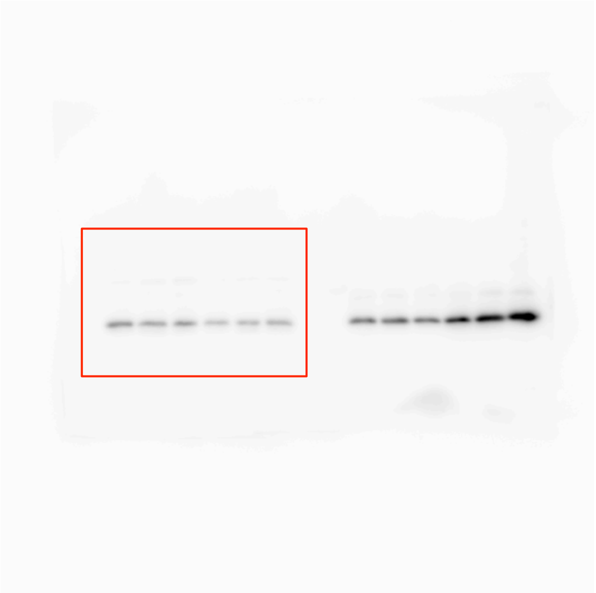

Figure 6C: total PRDX4

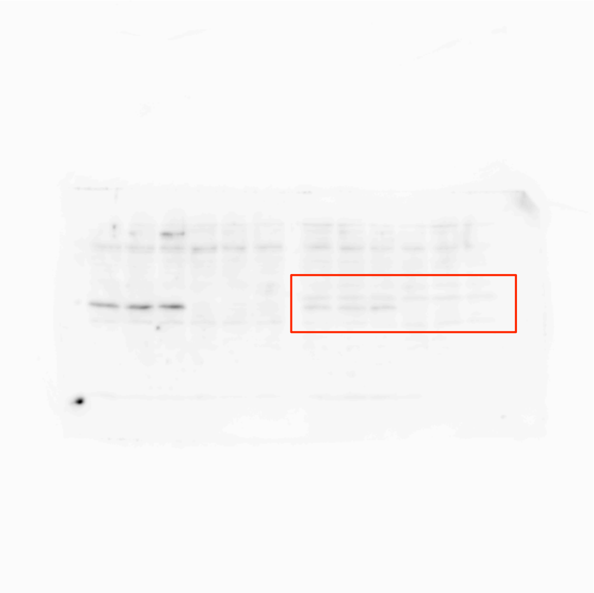

Figure 6C: PRDX4t specific

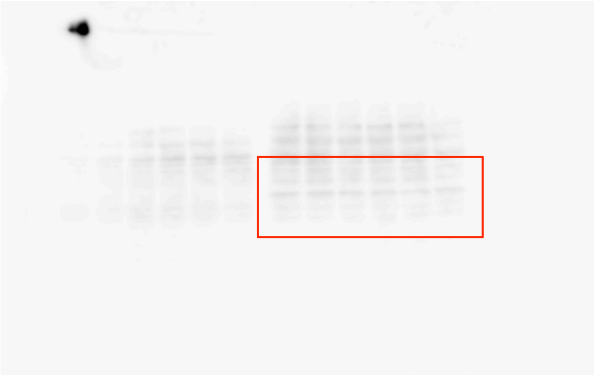

Figure 6C: PRDX6

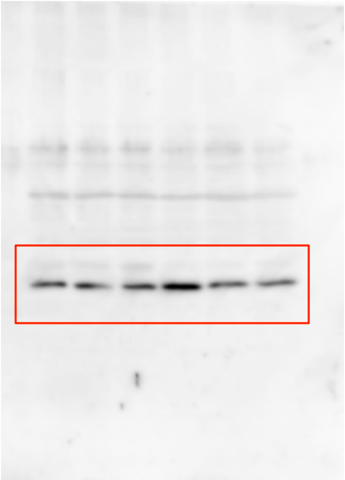

Figure 6C: β-actin

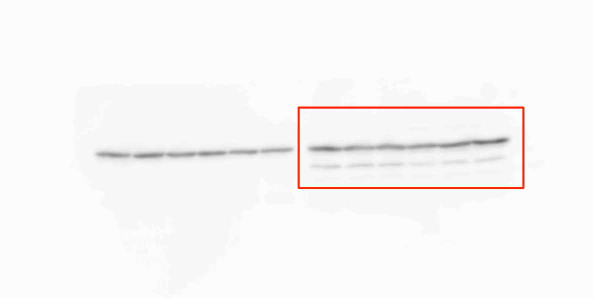

Supplementary Figure S12. Uncropped images of western blot presented in Figure 6C.

Figure S1: β-actin, Prdx4, Prdx4t

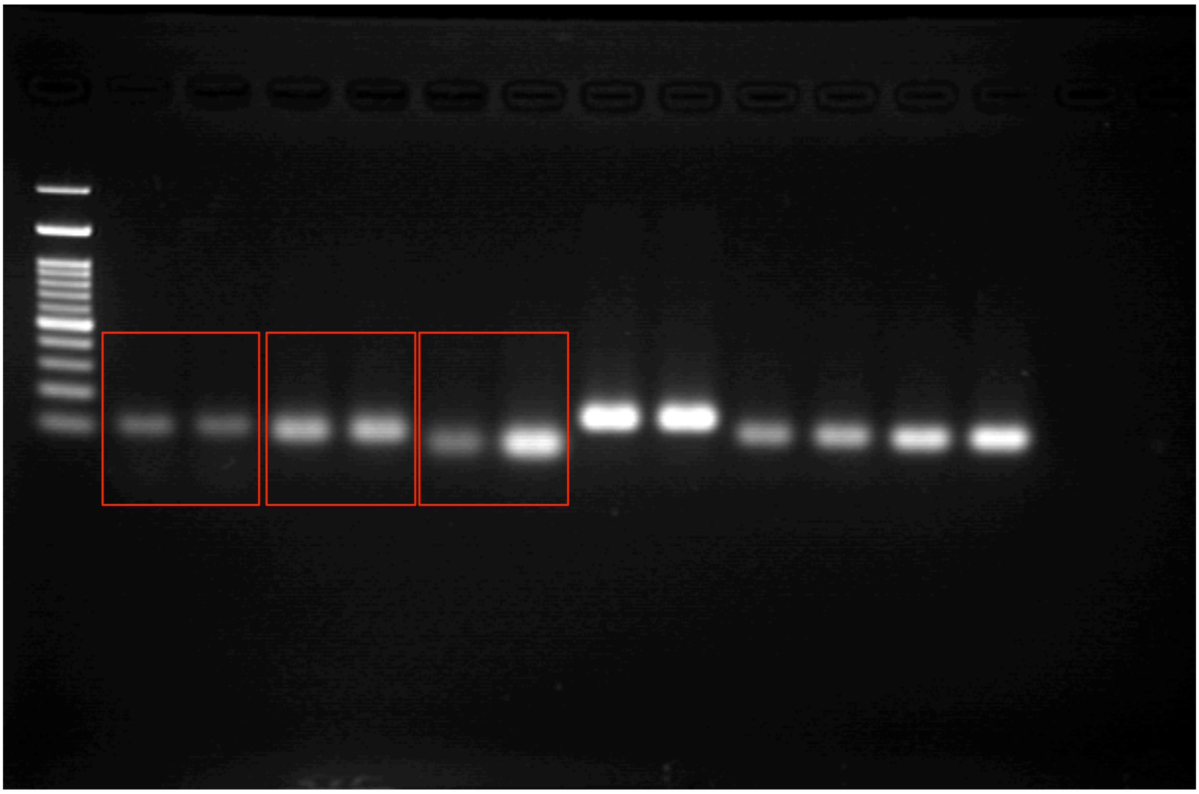

Supplementary Figure S13. Uncropped pictures of the agarose gel staining shown in the manuscript in Supplementary Figure S1.

Figure S3: GPX4

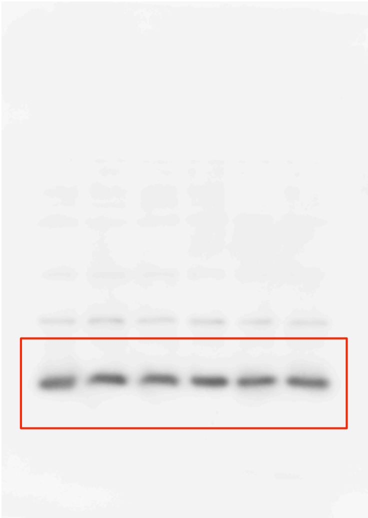

Figure S3: PRDX6

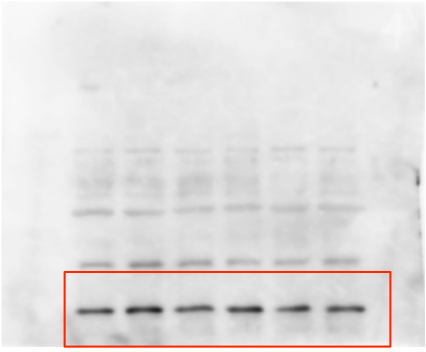

Figure S3: total PRDX4

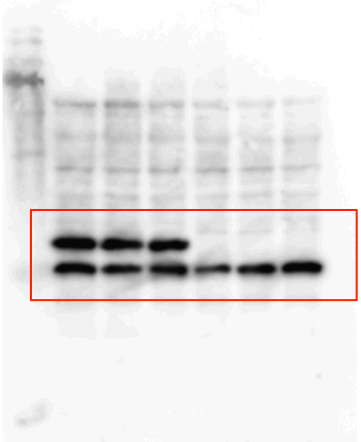

Figure S3: PRDX4t specific

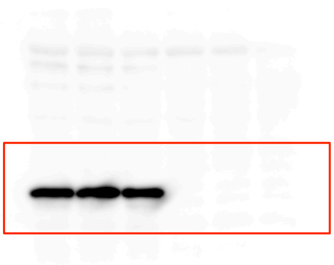

Figure S3: β-actin

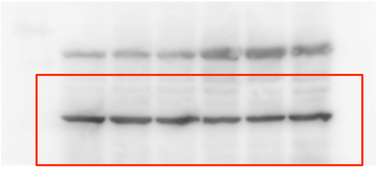

Supplementary Figure S14. Uncropped images of western blot presented in Supplementary Figure S3.

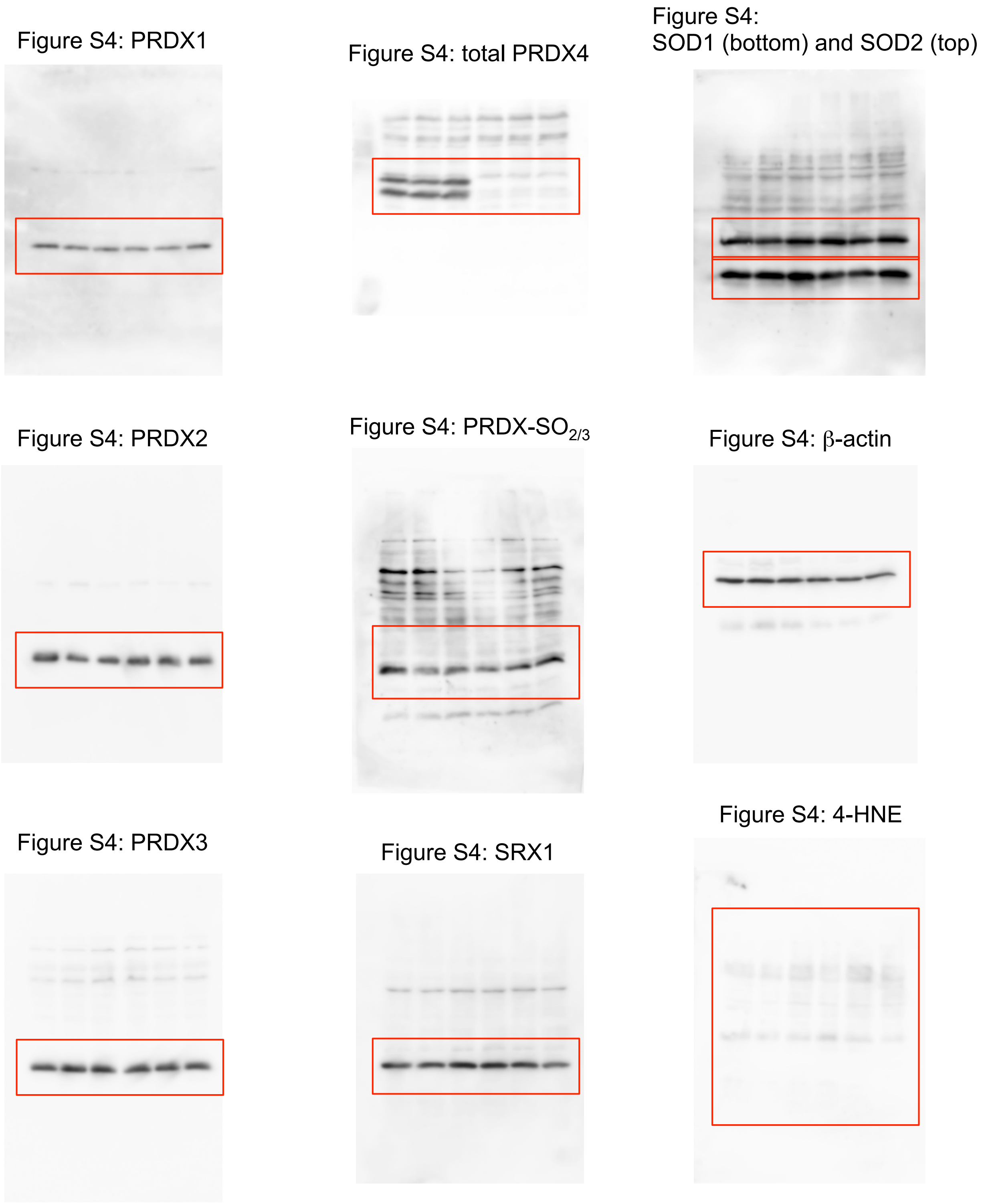

Supplementary Figure S15. Uncropped images of western blot presented in Supplementary Figure S4.
