# Supplementary material for: Testis-specific peroxiredoxin 4 variant is not absolutely required for spermatogenesis and fertility in mice
Source: Sci Rep. 2020 Oct 21;10:17934. doi: 10.1038/s41598-020-74667-9 (PMC7577974; doi:10.1038/s41598-020-74667-9)
Supplement: Supplementary file 2 — Supplementary Information 2. [file 41598_2020_74667_MOESM2_ESM.docx]

**Supplementary Table S1.** RNAs used for generating knockout mice.

|  | **RNA sequence (5’ to 3’)** |
| --- | --- |
| crRNA for alternative exon 1 | GAUCACCGUUGCCGUUCACGGUUUUAGAGCUAUGCUGUUUUG |
| crRNA for exon 2 | UGUGAUUAACGGAGAAUUCAGUUUUAGAGCUAUGCUGUUUUG |
| tracrRNA | AAACAGCAUAGCAAGUUAAAAUAAGGCUAGUCCGUUAUCAACUUGAAAAAGUGGCACCGAGUCGGUGCUUUUUUU |

Each crRNA was designed by appending the S. pyogenes crRNA repeat sequence GUUUUAGAGCUAUGCUGUUUUG to the 3’ end, which ensures base pairing to the tracrRNA.

**Supplementary Table S2.** Primers and PCR conditions used for genotype validation of CRISPR/Cas9-derived mutant mice.

|  | **Forward primer (5’ to 3’)** | **Reverse primer (5’ to 3’)** | **Annealing** | **Elongation** | **Band size** (bp) |
| --- | --- | --- | --- | --- | --- |
| *Line 37* | AATTTCAAACTTAGAGCCACAGC | CCTTCCCTAGCCTCCTGCT | 60°C, 30 s | 72°C, 30 s | WT: 182 / KO: 163 |
| *Line 38* | TGGATCACCGTTGCCGTTCA | CTCTTCGCTTTCTGGAGGTC | 60°C, 30 s | 72°C, 30 s | WT: 179 |
|  | TGGATCACCGTTGCCGTTCT |  |  |  | KO: 180 |
| *Line 80* | TGTGATTAACGGAGATCAAGG | TCCTTCTCTCAGCTTGCCTTA | 60°C, 30 s | 72°C, 30 s | DKO: 203 |
| *Line 81* | CTAAAACAGTCCTTGGCCATTC | CAAATCCAGTGGGTAGAAGAAAA | 60°C, 30 s | 72°C, 30 s | WT: 185 / DKO: 174 |

**Supplementary Table S3.** Primers used for RT-PCR.

| **Gene** | **Species** | **Forward Primer (5’ to 3’)** | **Reverse Primer (5’ to 3’)** | **Annealing** | **Elongation** | **Band size** (bp) |
| --- | --- | --- | --- | --- | --- | --- |
| *Prdx4* | Mouse | AAATGAGTGCCACTTCTACGC | TTGCTTAGATGCAGGGAGTG | 58°C, 30 s | 72°C, 30 s | 90 |
| *Prdx4t* | Mouse | CCAGAAAGCGAAGAGTTACAGG | TCCTTCCCAATAAGGTGCTG | 58°C, 30 s | 72°C, 30 s | 63 |
| *β-actin* | Mouse | TGAGGAGCACCCTGTGCT | ACATGGCTGGGGTGTTGAAG | 58°C, 30 s | 72°C, 30 s | 104 |
